# Editorial Note: Viral-mediated activation and inhibition of programmed cell death

**DOI:** 10.1371/journal.ppat.1011712

**Published:** 2023-10-10

**Authors:** The PLOS Pathogens Editors

Following the publication of this article [1], it was noted that there are potentially multiple errors in the reference list.

The authors confirmed a number of errors and are currently reviewing the full reference list. They agreed to revise the article to correct these errors, and they indicated that this may take 3 months or more.

Therefore, the *PLOS Pathogens* Editors issue this interim Editorial Note to notify readers of the concern regarding errors in the reference list.

## References

[1] Verburg SG, Lelievre RM, Westerveld MJ, Inkol JM, Sun YL, Workenhe ST (2022) Viral-mediated activation and inhibition of programmed cell death. PLoS Pathog 18(8): e1010718. 10.1371/journal.ppat.1010718 35951530 PMC9371342

